# Uniting the Role of Endophytic Fungi against Plant Pathogens and Their Interaction

**DOI:** 10.3390/jof9010072

**Published:** 2023-01-03

**Authors:** Shazia Akram, Ayesha Ahmed, Pengfei He, Pengbo He, Yinglong Liu, Yixin Wu, Shahzad Munir, Yueqiu He

**Affiliations:** 1 State Key Laboratory for Conservation and Utilization of Bio-Resources in Yunnan, Yunnan Agricultural University, Kunming 650201, China; 2 College of Agronomy and Biotechnology, Yunnan Agricultural University, Kunming 650201, China

**Keywords:** endophytes, fungus, phytopathogens, microbial interaction, biocontrol

## Abstract

Endophytic fungi are used as the most common microbial biological control agents (MBCAs) against phytopathogens and are ubiquitous in all plant parts. Most of the fungal species have roles against a variety of plant pathogens. Fungal endophytes provide different services to be used as pathogen control agents, using an important aspect in the form of enhanced plant growth and induced systemic resistance, produce a variety of antifungal secondary metabolites (lipopeptides, antibiotics and enzymes) through colonization, and compete with other pathogenic microorganisms for growth factors (space and nutrients). The purpose of this review is to highlight the biological control potential of fungal species with antifungal properties against different fungal plant pathogens. We focused on the introduction, biology, isolation, identification of endophytic fungi, and their antifungal activity against fungal plant pathogens. The endosymbionts have developed specific genes that exhibited endophytic behavior and demonstrated defensive responses against pathogens such as antibiosis, parasitism, lytic enzyme and competition, siderophore production, and indirect responses by induced systemic resistance (ISR) in the host plant. Finally, different microscopic detection techniques to study microbial interactions (endophytic and pathogenic fungal interactions) in host plants are briefly discussed.

## 1. Introduction

Endophytes are a fascinating group of microorganisms associated with internal plant tissues or related organs. The endophyte term was first proposed by German scientist Heinrich Anton De Barry [1] to define fungi and bacteria that colonize plant tissues without causing any harm to their host [2]. Endophytic fungi protect the plant through well-organized direct and indirect mechanisms. They are ubiquitous in the plant kingdom and have been isolated from almost all plant parts. Endophytic fungi build a mutualistic relationship with its host plant [3,4,5]. These associations lead to plant growth promotion [6], pathogen inhibition [3], soil pollutant removal [7], and improving tolerance to abiotic stress such as salinity, drought, and extreme temperature [8,9], all of which are prevailing threats to agricultural food production [10].

Endophytes can survive in plant tissues for a long time and protect the plant from biotic and abiotic stress [11]. Various fungal entophytes have been found in many important plants such as tobacco [12], tomato [13], wheat [14,15], and banana [16,17]. They establish interspecies interactions through direct mechanisms such as competition, parasitic, and antimicrobial effects through the production of primary and secondary metabolites, enzymes or volatile compounds and indirect mechanisms (induced resistance), protecting from pathogen invasion [18,19,20]. However, endophytic fungi colonizing in healthy plant tissues may also switch from non-pathogenic to pathogenic mode when the plant is under stress [2].

Fungal endophytes perform multiple functions including protection against phytopathogens [21], herbivorous insects [22], and plant parasitic nematodes [23]. They are natural enemies of plant pathogens and act as microbial biocontrol agents (MBCAs) to control plant diseases without harming the host plant, human, and environmental health. Above all, the risk of evolution of pathogen resistance is likely impossible compared to agrochemicals [24]. Microorganisms including fungi, bacteria, viruses, and nematodes are being successfully used as MBCAs worldwide [25]. Similarly, fungal endophytes including entomopathogenic fungi are very important in all MBCAs due to their wide host range, diverse antagonistic mechanisms against sap sucking pests such as mosquitoes and aphids [26,27,28] as well as fungal pathogens of different field crops [29,30,31]. A simple delivery method, enhanced formulation, already characterized pathogenic strains, overexpression of endogenous toxins, and uncomplicated engineering techniques make these fungal endophytes suitable candidates for agricultural applications [32,33,34].

Fungal endophytes are a potential source of new natural products for agricultural development. In this review, we highlight the important endogenous role of several endophytic fungi in the biocontrol of pathogenic fungi with some of the best-studied examples. In addition, we also explain how fungal entophytes serve as a route to achieve long-term sustainable agricultural development.

## 2. Classical Identification Strategies for Endophytic Fungi

Endophytic fungi are isolated from plant parts through culture dependent and culture independent methods [35]. The culture dependent method is commonly used for the identification of fungal endophytes [36,37] and to assess the diversity of endophytic species [38]. The detection accuracy depends on abiotic, biotic, and experimental factors [39,40]. The data of potential fungi have been compiled by referring to host indices, culture collections, herbs, and fungal monographs. The standard classification manuals (i.e., “Manual for fungi”) are most commonly used for the isolation and identification of fungi [41].

The physical inspection of field plants and the collection of fungal samples from host plants are very effective methods for studying endophytes [42]. Observing the effects of endophytes on their host can reveal important clues about their life cycle, mode of transmission, identification, and association with their host [43,44,45]. The fructose-derived cultures and DNA reference specimens are important and are used to identify the endogenous state of organisms. The staining technique [46], culture-based [47], and culture independent [35] detection methods have been successfully used to study asymptomatic endophytic fungi in healthy plant tissues. The recovery and detection of endophytic fungi is undertaken by dissecting plant organs into small pieces, sterilizing their surfaces, and placing those pieces on a nutrient-rich agar medium [39,48]. The size of the tissue fragments used for endophyte isolation is negatively correlated with the estimated value of endophytic species richness [49]. Important issues related to endophyte isolation are fungal growth on the agar medium, the quantity of samples and plant analysis patterns [50,51]. Several studies have suggested that biotrophic fungi infecting live plant tissues can only be detected by direct observation on the surface of the host, and these fungi have always resisted attempts to be cultured in vitro [52].

So far, the composition and diversity of endophytic fungal communities of all examined plant species have been evaluated through culture-based methods [53], while morphological characters coupled with molecular analysis techniques have been used to identify non-culturable endophytic fungi [54]. The detection of fungal DNA from plant tissues through molecular methods have revealed several dimensions of a fungal endophytic community, which was impossible through cultivation methods [35,55]. Several endophytic communities have been detected by the fingerprinting technique and amplification of ITS amplicons from the extracted DNA [56,57]. A big difference in the dominant fungal communities was observed between the culture-dependent and culture-independent methods. The detection of different taxa relies upon the detection method [58]. Fungi represent many ecological functions such as endophytes, pathogens, and saprobes [59]. In this case, the taxa of fungi that the DNA detection method perceives are similar to the combination of direct observation and culture methods [60]. Certain endophytes are uncultivable, so culture-independent approaches such as next generation sequencing (NGS) can help in a better understanding of their ecology and distribution [35], and revealed all frequently detected fungal genera from a culture-dependent approach that had a relative abundance higher than 5%. Many scientists have realized that 99% of prokaryotic microorganisms may not be cultivated [61].

Many researchers believe that the study of fungal endophytes based on culture can only estimate the diversity of the misinterpreted endophytic community and taxonomic composition [62]. Therefore, culture-independent methods such as DNA cloning [63], terminal restriction fragment length polymorphism (TRFLP or sometimes T-RFLP [58,64,65] and denaturing gradient gel electrophoresis (DGGE) [66,67] are considered more efficient for resolving fungal diversity. However, despite some limitations and the need for further improvement, these methods [68,69] have gained much popularity compared to culture-based techniques as a means of assessing the overall diversity and composition of endophytic fungal communities [70,71].

## 3. Mechanisms of Fungal Endophytes

**Figure 1:**
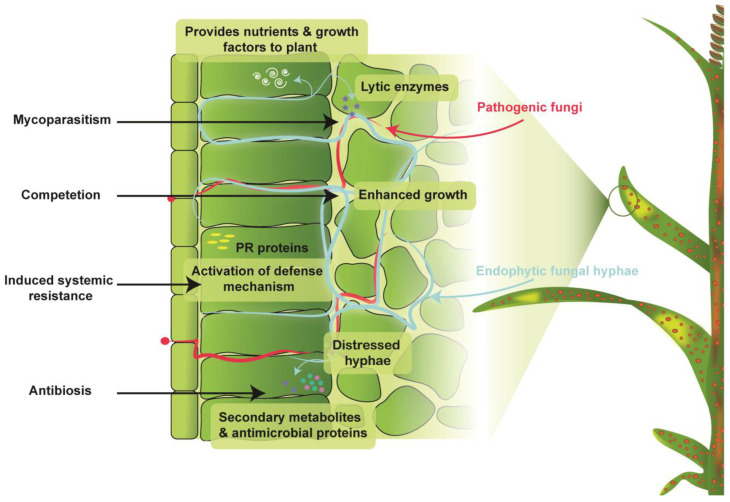
Mechanisms of fungal endophyte interaction with fungal plant pathogens.

Endophytic microorganisms improve the adaptability of plants by employing different mechanisms of action. Endophytic fungi commonly adopt mechanisms including pathogen inhibition directly through competition, antibiosis, and mycoparasitism while indirectly through the induction of resistance (Figure 1), thereby activating the plant’s defense system to resist the disease [72].

### 3.1. Competition

Competition is a potential mechanism adopted by endophytes to prevent pathogens from colonizing in host tissues [73]. Endophytes have the ability to colonize locally or systematically in plant tissues by utilizing the available nutrients and occupying the space, thus creating an unsuitable environment for pathogen growth [74,75]. Mechanisms used by endophytes for competition usually function in conjunction with other mechanisms, rather than operating individually [76]. The mechanisms mostly have direct effects, thus for better pathogen antagonism, an endophyte must colonize in the plant systemically [77]. For instance, the foliar application of an endophytic mixture from cacao leaves significantly reduced the *Phytophthora* infection through competition. However, some strains were also found to produce active metabolites, which suggests that competition may not be the only mechanism for disease control [78]. As *Heteroconium chaetospira* endophyte colonizes in roots, but it could not effectively suppress the clubroot infection in oil seed rape [79]. The studies suggest that endophytes operating solely with competition as a biocontrol mechanism may not be effective in high pathogen load [80]

### 3.2. Induction of Resistance

Endophytic fungi are known to activate plant defense against pest or pathogen attack. This activation event improves the plant defense responses against future pathogen attack, enabling it to perform more efficiently; this state is also known as induced systemic resistance or defense priming [81]. Induced systemic resistance (ISR) is commonly regulated by ethylene or jasmonic acid, which does not include the upregulation of pathogenicity-related (PR) proteins [82]. On the other hand, systemic acquired resistance (SAR) is generally associated with pathogen infection and is mediated by salicylic acid, further leading to the accumulation of the PR protein [83]. These PR proteins include various enzymes such as chitinase and beta 1,3-glucanase, which directly dissolve the invading pathogen cells and strengthen the cell wall boundary to establish the ability to resist the infection and cell demise [21]. However, induction of systemic resistance by endophytes can also be associated with augmentation of pathogenesis related genes [84]. *Fusarium solani*, isolated from tomato plants, was reported to promote ISR against foliar pathogen *Septorialyco persici* by activating the PR, PR7, and PR5 genes in the root [85]. In another study, non-pathogenic mutant of *Colletotrichum magna* strains induced resistance in *Citrullus lanatus* and *Cucumis sativus* plants by producing large amounts of peroxidase, phenylalanine ammonia lyase enzyme, and lignin deposition, which helped protect the plants from infection caused by *C. orbiculare* and *F. oxysporum* [19,86,87]. Furthermore, the contact of *Neotyphodium lolii* with different pathogens decreased leaf lesions and necrotic spots disease symptoms by enhancing superoxide dismutase and peroxidase activities in the host plant [88]. However, endophytes have different modes of action such as competition between the endophytes and pathogen and the induction of plant resistance by producing various metabolites, but more research is still required to understand this mechanism [78]. Microbial metabolites produced in the host also play an important role in providing resistance against the invading pathogens. These kinds of metabolite inducers are specific to fungi and are able to induce the plant defense response. Pathogen associated molecular patterns (PAMPs) and microbial associated molecular patterns (MAMPs) play significant roles as compounds of microbial origin that are easily recognized by plants. In this regard, the fungal cell wall components chitin and β-glucans are the important MAMPs recognized by plants [89]. 

### 3.3. Mycoparasitism

This is another important mechanism used by endophytes to protect the host ecology by directly attacking the identified pathogen or its propagules [90]. Endophytic fungi penetrate in the hyphae of pathogenic fungi and destroy the cell walls of pathogens by lysing cells. This mechanism can be divided into the following four steps. The first and second steps involve recognition of fungal pathogens and chemotropic growth of the endophytic fungal mycelium toward pathogenic fungi, respectively. The third and fourth steps involve the direct physical contact of endophytic fungi with pathogenic fungi and penetration into the target fungal cells, which result in cell wall degradation [91]. Different species of *Trichoderma* involved in biocontrol were found to penetrate the hyphae of pathogenic fungi *Rhizoctonia solani* and kill them [92]. Similarly, endophytic fungi isolated from common reed inhibited the growth of soil borne fungal pathogens by coiling around their hyphae, causing degradation of the hyphal cytoplasm after penetrating the cells. The degradation of fungal hyphae involves the secretion of various cell wall degrading enzymes by endophytic fungi [93]. Several fungal antagonists such as *Acrodontium crateriforme*, *Acremonium alternatum*, *Ampelomyces quisqualis*, and *Gliocladium virens* are among the few fungi with the ability to parasitize powdery mildew pathogens [94].

### 3.4. Antibiosis 

Endophytes produce various secondary metabolites, some of which have antifungal and antibacterial properties and help to inhibit the growth of plant pathogenic microorganisms [95,96]. Many metabolites (i.e., peptides, flavonoids, quinones, phenols, alkaloids, steroids, polyketides, and terpenes) with antimicrobial activity have been reported from endophytes [95,97,98]. However, there is a dire need to explore the secondary metabolites produced by endophytic fungi for commercial purposes [3,99]. Associations between the host and endophytes encourage the production and secretion of metabolites with diverse functions such as pathogen inhibition [100,101]. It is also known that certain endophytes share similar gene clusters using the same precursors for the co-production of active metabolites [102,103,104]. Many studies have reported that many endophytic strains cannot independently produce compounds involved in host plant resistance against pathogen attack [105,106]. Loper and Ishimaru [107] identified two components of *P. ultimum* suppression on cotton using an antifungal minus mutant of *P. fluorescens*. They claimed that antibiotic synthesis was the most important factor, with seed and root colonization coming in second. The use of enzymes in biocontrol makes it difficult to distinguish between parasitism and antibiosis. An antagonist’s production of a cell wall destroying enzyme, for example, would almost certainly be involved in parasitism and antibiosis at the same time. Antibiosis may be the only effect of other enzymes. For example, under field conditions, the *Talaromyces flavus* isolate Tfl (anamorph *Penicillium dangeardii*, also known as *P. vermiculatum*) suppresses verticillium wilt of eggplant (Brunner et al., 2005) and has the ability to suppress the verticillium wilt of potato [108]. In the soil, *Talaromyces* is a strong competitor [109].

Furthermore, this antagonist produces a chemical that kills *V. dahliae* micro sclerotia *in vitro* and in soil [110]. The identification of this molecule as glucose oxidase was aided by the revelation that the acetone precipitable portion interacted exclusively with glucose [111]. This process produces hydrogen peroxide, which kills the pathogen’s sclerotia. The addition of glucose or glucose oxidase to soil does not kill the microsclerotia, but the addition of both glucose and glucose oxidase reduces the quantity of live microsclerotia buried in soils. Hydrogen peroxide has also been linked to the antibiotic lactobacillin [112].

## 4. The Interaction of Fungal Endophytes and Pathogens in Different Host Plants 

Plant pathogens pose a major threat to food security and ecosystem stability [113]. It is estimated that pathogen attacks will reduce about 30–50% of global crop production, leading to an increase in poverty and malnutrition [114]. Among the phytopathogens, fungi are considered as one of the most destructive pathogens in agriculture [115]. The research on the biological control of phytopathogens is a relatively emerging field, however, several studies have supported the role of endophytes in pathogen inhibition. Fungal endophytes play an important role in plant–pathogen interactions. It has been observed that they can employ multiple mechanisms to inhibit pathogens (i.e., some endophytes induce the plant defense system against pathogen invasion), and some produce antimicrobial compounds that directly inhibit pathogen growth, while others compete for niche and nutrients (Table 1) [19,116]. The endophytic biocontrol strains of *Trichoderma* and *Sebacinales* spp. have been known to control many root, foliar, and fruit pathogens, alleviate various abiotic stresses, physiological stresses (seed age) as well as enhance nutrient absorption. These endophytic strains also increase the photosynthesis and respiratory activities of plants. These functions are associated with their ability to reprogram plant gene expression, possibly by activating some of the universal plant pathways [117].

Finally, some parasites of phytopathogens in the host plant are known to act as endophytes and have various biological functions. Endophytic fungi in host plants (i.e., in tobacco), not only promote the growth but also enhance the resistance to biotic and abiotic factors [118,119,120]. The endophytic fungi in the host plant may provide biological protection to the host plant from pathogens, pests, and even domestic herbivores. Several species of endophytic fungi were investigated, including *Aspergillus niger*, *A. flavus*, and *Penicillium* where isolated endophytic flora of the *Aspergillus genus* was the abundant [121].

The fungal endophytes isolated from the healthy tissue of cacao trees were tested to antagonize the pathogens *Monilio phthoraperniciosa*, *M. phthoraroreri*, and *Phytophthora palmivora* of cacao. The mechanism for antagonism was solely competition for the substrate. The endophytic fungi reduced the pod loss caused by *Phytophthora* spp. and the sporulation *of M. roreri*, which support the fungal endophyte’s potential as a biocontrol agent [122]. Similarly, the colletotric acid, isolated from the *Colletotrichum gloeosporioides* endophyte, inhibited the pathogenic fungus *Helmintho sporium sativum* [123]. Muscodor (*Muscodoralbus*) is an endophytic fungus isolated from the Cinnamomum tree (*Cinnamomum zeylanicum*) and produces volatile compounds, esters, alcohols, lipids, ketones, and acids, which effectively inhibit fungi and bacteria [124]. The volatile compounds secreted by *M. albus* inhibited and killed a variety of storage pathogens [125]. The 132 endophytic fungi isolated from 21 banana varieties were recovered with common endophytic strains of *Fusarium* (28 strains), *Cephalosporium acremonium*, *Verticillium* wilt, and *Trichoderma* under greenhouse conditions and tested against *Radopholus similis* [126]. The isolates of *Epicoccum nigrum*, *Trichoderma viride*, *Sclerotinia sclerotiorum*, *Fusarium tricinctum*, *Cytospora* spp., and *Alternaria alternate* significantly controlled *Diplodia corticola*, a causal agent of wilting, vascular necrosis, and the death of various oak trees. These observations indicate that interaction between endophytes in plants and *D. corticola* is very complex and requires further study [127]. 

*Trichoderma* spp. produced hydrolytic enzymes when they invaded the mycelium of *Fusarium solani*, *Rhizoctonia solani*, and *Sclerotinia sclerotiorum* and inhibited the growth of pathogens [128]. The DNA-dependent SSCP analysis of 16S rDNA/ITS sequences determined the effect of *Trichoderma* on local root-related microbial communities, and as a result, three *Trichoderma* strains originally isolated from *Rhizoctonia sclerotia* were selected as promising BCA [92]. Endophytic fungal communities including *Lasiodiplodia*, *Acremonium*, *Cladosporium*, *Blastomyces*, *Botryosphaeria*, *Colletotrichum*, *Geotrichum*, *Cordyceps*, *Diaporthe*, *Fusarium*, *Gibberella*, *Gliocladium*, *Nectria*, *Monilochoetes*, *Pestalotiopsis*, *Phomopsis*, *Pseudo fusarium*, *Pleurotus, Rhizopycnis*, *Syncephalastrum*, *Verticillium*, *Xylaria*, and *Trichoderma* were found to be potential antagonists to *Clostridium perennial*. Among all endophytes, *Gliocladium catenulatum* reduced witch broom disease incidence more than 70% [129]. The arbuscular mycorrhizal fungi (AMF) significantly reduced rust and powdery mildews by inducing systemic resistance [130]. The *Lecanicillium* spp. controlled various plant pathogens by adopting a parasitism mechanism [112]. The *Cordana* spp., *Nodulis porium* spp., and mixtures of endophyte and *Fusarium verticillioides* significantly inhibited important fungal pathogens (i.e., *Colletrotrichum*, *P. monticola*, and *Ustilago maydis*) [55].

**Table 1:** Fungal endophytes as biological control agents against pathogenic fungus.

No	Endophytic Fungi	Pathogenic Fungus	Disease Control	References
1	*Aspergillus flavus*, *A. niger*, *A. terreus. Aspergillus* spp., *Penicillium sublateritium*, *Penicillium* spp. from *FCV tobacco*	Pathogenic fungi		[121]
2	*Colletotrichum gloeosporioides*, *Clonostachys rosea* and *Botryosphaeria ribis*	*Monilio roreri, Phytophthora* spp.	Black pod rot	[122]
3	*Colletotrichum gloeosporioides*	*Helminthosporiu sativum*		[123]
4	*Seimatoantleriumtepuiense* (produced fungal paclitaxel)	*Pythium* spp. *Phytophthora* spp.		[131]
5	*Muscodoralbus*, *Trichoderma*	*Candida albicans*, *Rhizoctonia solani*, *Pythium ultimum*, and *Fusarium oxysporum*.		[124]
6	*Muscodor albus*	*Penicillium expansum* *, Botrytis cinerea, Monilinia fructicola*	Fungal decay of apples and peaches	[125]
7	Banana endophytic fungi (strains of *Fusarium*, *Acremonium*, *Verticillium*, and *Trichoderma*)	*Radopholus similis*		[126]
8	*Trichoderma viride*, *Epico cumnigrum*, *Fusarium tricinctum*, *Alternaria alternata*, *Sclerotinia sclerotiorum*, *Cytospora*	*Diplodia corticola*	Cankers, vascular necrosis, and dieback	[132]
9	*Trichoderma asperellum T203*	*Pseudomonas syringae* pv. *lachrymans*		[133]
10	*Trichoderma harzianum*, *T. asperellum*	*Fusarium solani*, *Rhizoctonia solani*, and *Sclerotinia sclerotiorum*		[128]
11	*Rhizoctonia sclerotia* (*Trichoderma reesei* and *T. viride*)	*Rhizoctonia solani*		[92]
12	*Gliocladium catenulatum*	*Crinipellis perniciosa*	Witches’ broom Disease of cacao	[129]
13	*Arbuscular mycorrhizal*	*Phytophythora*, *Fusarium, Sclerotium*, *Verticillium*, *Aphanomyces*		[130]
14	*Cordana* spp., *Nodulisporium* spp., mixtures of endophytes, *Fusarium verticillioides*	*Colletrotrichum*, *P. monticola*, *Ustilago maydis*	Witches broom, white pine blister rust, smut disease	[134]
15	*Sebacinales* spp.	Numerous foliar, root, and fruit pathogens		[117]
16	Root endophytic fungi	*Verticillium dahliae*	Verticillium wilt in egg plant	[135]
17	Foliar endophytic fungi from *Hevea brasiliensis*	*Microcyclusulei*	South American leaf blight	[136]
18	*Verticillium lecanii* (chrysanthemum host)	*Puccini horiana*	White rust	[137]
19	Endophytic fungus from the Chinese medicinal plant *Arisaema erubescens*	*Fusarium oxysporium*, *Rhizoctonia solani*, *Colletotrichum gloeosporioides*, and *Magnaporthe oryzae*		[138]
20	*Cladosporium* spp., Endophyte of *Quercus variabilis*	*Trichophyton rubrum*, *Candida albicans, Aspergillus niger, Epidermophyton floccosum*, *Microsporum canis*		[139]
21	*Chaetomium globosum*, endophyte of wheat	*Pyrenophora* spp.	Tan spot disease of wheat	[140]
22	Endophytes (isolated from *Theobroma cacao* L.)	*Phytophthora* spp.		[141]
23	*Acremonium strictum*	*Helminthosporium solani*	Silver scurf potato tuber disease	[142]
24	*Beauveria bassiana* (Cotton seed)	*Rhizoctonia solani*, *Pythium myriotylum*, and *Thielaviopsisbasicol*	Seedling diseases	[29]
25	*Beauveria bassiana*, *B. brongniartii*	*Pythium ultimum*, *P. debaryanum*		[143]
26	*Beauveria bassiana* (cotton and tomato seed)	*Rhizoctonia solani*, *Pythium myriotylum*, and *Xanthomonas axonopodis* pv. *malvacearum*	Damping off seedlings and root rot (bacterial blight).	[144]
27	*B. bassiana Vuillem* in (tomato seedling)	*Pythium myriotylum*	Pythium damping-off	[145]
28	*B. bassiana strains* (sugar cane)	*Colletotrichum falcatum*	Red rot of sugar cane	[146]
29	*Metarhizium robertsii*	*Fusarium solanif* sp. *phaseol*		[147]
30	*Beauveria bassiana*	*Fusarium graminearum*, *F.avenaceum*, *Aspergillusparaziticus*, *F.oxysporum*, *Alternaria tennus*, *F. moniliforme*		[148]
31	Yeasts	Soil-borne fungal root pathogens		[149]

The 123 fungal isolates including *Phialocephala fortinii* and *Radicis atrovirens* isolates are commonly isolated from melon, eggplant, tomato, Chinese cabbage, and strawberry bait plants. The mechanism by which these endophytes confer resistance is unclear, but laboratory tests have shown that they have significant potential as BCAs [135]. Endophytic fungi isolated from *Hevea brasiliensis* (rubber tree) inhibited *Microcyclus ulei*, a causal agent of South American leaf blight (SALB) [136].

Similarly, *Verticillium lecanii* parasitized the spores and fruiting bodies of *Puccinia horiana*, causing chrysanthemum white rust [150]. The fungal endophytes, *Clado sporium* spp. produced santibiotic metabolites, which inhibited the growth of pathogenic fungi including *Trichophyton*, *Candida albicans*, and *A. niger* [139]. During the experiment, a protective effect of endophytes on the plants’ pathogenic fungi after inoculation produced an antifungal substance, which induced plant defense mechanisms rather than direct antagonism. The same is the case with endophytic fungi *Chaetomium* in wheat, where the fungi reduced the severity of leaf diseases caused by *Pyrenophora* spp. [140]. The leaf diseases caused by *Phytophthora* spp. were significantly reduced when host plant leaves were inoculated with a mixture of six endophytes isolated from *Theobroma cacao* [78]. Infection by endophytes may alter the biochemistry of plants in a way that induces defense mechanisms against pathogens. The endophytes *Acremonium strictum* isolated from *Dactylis glomerata* acted as a mycoparasite of the *Helminthosporium solani* causal agent of potato tuber silver scurf disease and significantly reduced pathogen inocula (Table 1) [142].

*Beauveria bassiana* in cotton has endophytic biocontrol activity against *Rhizoctonia solani* in tomato seedling [29]. For *B. bassiana* and *B. brongniartii*, both species were antagonistic to *P. debaryanum*, and *Septoria nodorum* while *P. irregulare*, *Pleospora betae*, *Rhizoctonia solani*, and *P. exigua* var. *foveate* showed resistance to *Beauvaria* spp. Both endophytic fungi induced the lysis of phytopathogenic fungi jointly when cultured on agar medium. *B. brongniartii* colonized more effectively than *B. bassiana* and inhibited the growth of phytopathogenic fungi [150]. The application of *B. bassiana* on seed resulted in endophytic colonies in tomato and cotton seedlings, which prevented *Rhizoctonia solani* and *Pythium myriotylum* infection, a causal agent of damping off and root rot. The degree of disease control depends upon the *B. bassiana* conidial population density on the seed [144]. *Pythium* damping off causes severe losses in tomato crops grown in greenhouses and fields. Currently, there are no tomato varieties that are resistant to damping off. *Beauveria bassiana* isolates suppressed the damping off of tomato seedlings by inducing systemic resistance [145]. The entomopathogenic fungi produced chitinase enzymes, which are related to the inhibition of *Colletotrichum falcatum*, a causal agent of red rot disease of sugarcane [151]. The endophytic insect pathogenic fungus *Metarhizium robertsii* inhibited *F. solani* conidial germination [147]. The production of secondary metabolites by endophytic fungi is very important in the application of biotechnology. *B. bassiana*, as a crude extract at concentrations between 1200 and 1600 µg/mL, showed moderate antifungal activity against *Alternaria tenuis, Fusarium avenaceum*, and *F. graminearum* and the inhibitory effect was significant against *Aspergillus paraziticus*, *F. moniliforme*, and *F. oxysporum* [148].

Compared with filamentous fungal antagonists, yeast is little known as a biological control agent for soil-borne fungal plant pathogens. The potential antagonistic mechanism of yeasts against soil-borne fungal plant pathogens is expected to be similar to those antagonistic fungi towards leaf and fruit pathogens. Several yeasts have been recorded as endophytes in plants and a small portion of them have been recorded as promoters of plant growth [152]. Yeast reproduces rapidly, produces antibiotics, and degrading enzymes, which induce resistance and produce plant growth promoters. Various yeast genera have been used to control post-harvest diseases, especially those caused by fungal plant pathogens spread by fruits and soil [149].

## 5. Host–Plant Interaction with Endophytes 

The above studies showed that the results of certain pathogen invasion may depend on the endophytic fungal branches associated with the host plant. Therefore, the endogenous combination of a certain fungal species may correspond to a biological source with potential applications in the disease control of the following plant species. This indicates that the plant part of the disease cycle is shared by pathogens or endophytes. When fungus enters in a plant, it can act as an endophyte or pathogen, and in more cases, the fungi associated with plants can act as endophytes [153]. Endophytes successfully colonize a host tissue through establishing compatible host plant–microbe interactions [154]. When endophytes invade a host plant, the plant will recognize it and produce cross-talk signal molecules [155]. Later on, endophytes show a chemo tactic response to root exudates of the host plants [156,157]. The rich biomolecules, nutrients, and water are present in root exudates which attract the available microorganisms, but preferably mutually beneficial microorganisms including endophytes [102]. The plant’s immune system also plays an important role in allowing beneficial microorganisms to enter in plant tissues including fungal endophytes [158]. In addition, finding strategies for plants to distinguish beneficial microorganisms such as pathogens or endophytes is still a research problem [159]. Now that many endophytes and their genes have been identified, these endophytes and their genes can help to understand their behavior and mechanisms [102]. The plant can also induce the different genome expression in various endophytic microbes during their colonization [160,161]. The progress of symbiosis research shows that through nutritional monitoring, plants can identify whether invading microorganisms are beneficial or pathogenic [162,163]. The genotype of the host plant also significantly affects the endosphere microbiome community in host plants [164,165]. In some endophytes, in addition to local abiotic stress factors, it has also been found that the change of their lifestyle to a pathogenic state also depends on the genotype of the host (i.e., *Ramularia collocygni* can survive as an asymptomatic endophyte during the initial growth period, but in the later growth period, it switches to necrotic pathogen) [166,167]. At the ecological time scale, *Fusarium verticillioides* in maize plant can survive as endophytes or later become a latent pathogen that causes disease over a period of time [168]. However, external and endogenous factors that cause fungi to transform from endophytes to pathogens are not yet fully understood. In order to better understand the dynamics of endophytes, comparative studies are needed to find the gene expression and conditions (in plants and endophytes) in which the same microorganisms behave as symbionts or pathogens [102]. However, for endogenous lifestyles, a single mechanism or factor has not yet been determined, so further discoveries are needed.

## 6. Microscopic Techniques to Study Host–Plant Interaction with Endophytes 

**Figure 2:**
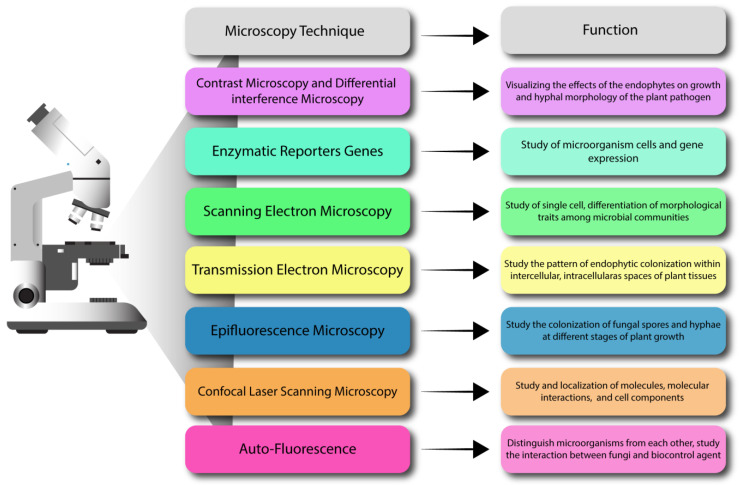
Microscopy techniques to study the interactions among plants, pathogens, and endophytes.

Endophytic fungi may exist in plants as mutualists, pathogens, or commensals, which neither harm the host plant nor provide any benefits [166]. The plant–microbe associations have drastic effects on the fitness of the host plant [169]. Thus, exploring these complex associations is indeed very important to fill the knowledge gap and harness the maximum benefits from beneficial endophytic fungi. The structure of fungi including hyphae, spores, and fruiting bodies can be observed through optical and electron microscopy [170]. The interactions between plant and endophytic fungi initiate from the attachment of fungal hyphae on the surface of plants, leading to infection, invasions, colonization in plant tissues, proliferation, sporulation expression of phenotypic traits, and spread to other individual plants [166,171,172,173]. Several approaches have been used to study the complex interactions between endophytes and plants. The genetics and enzyme activity measurement are powerful tools for discovering genes and traits related to these connections [174,175]. Presently, microscopic tools and molecular methods have contributed to new insights into the host–plant microbe interaction (Figure 2).

Molecular and microscopic techniques provide a better understanding of endophytic colonization within the host plant cell. When endophytes successfully colonize in host plant cells and develop intimate relationships, this may lead to information exchange at the cellular or molecular level, as illustrated in Table 2 [176]. Knowledge of the mechanism of endophyte colonization in host plants at the ultra-structural level can help to understand their efficiency as biological control agents [177]. A direct way of examining fungal endophyte colonization in the host cell is through microscopy. Although a light microscope is a convenient tool for observing plant–microbe interactions, it lacks a resolution to observe host–microbe interactions in detail. These microscopic studies provide evidence of endophyte colonization and isolation from the host plant cell. Microscopy has evolved continuously since the discovery of microorganisms, and modern microscopes can now solve highly complex problems. However, live microscopy of plants in a biotic and abiotic environment is still complex and has hardly been achieved (e.g., plant–environment microscopy tracks the interactions of *Bacillus subtilis* with plant roots across the entire rhizosphere). 

Electron microscopy provides a comprehensive understanding of endophyte colonization in host plant cell at the superstructure level. Electron microscopic studies have revealed pathogen morphological damage and deformities caused by the endophyte culture extract [178]. Visualizing the interactions between microorganisms and plants at the cellular level provides detailed basic principles of how endophytes work [102]. Methods for the detection of endophytes in host tissues have not been well-documented in the scientific literatures. Nowadays, confocal laser scanning (CLSM) is a critical step in studying these elusive organisms [179]. The main advantage of using CLSM over ordinary microscopy is that CLSM has a very high specific resolution and much higher contrast than conventional microscopy [180]. Basically, laser scanning confocal microscopy is a technique superior to conventional microscopy, which can provide a higher resolution of endophytic fungi in dark septum against a background of the cortical host plant cell [180]. The highly sophisticated microscope tools and the optimization of auto fluorescent proteins have made important contributions to understand how these microorganisms interact with plants [177]. These reasonable microscopy tools will promote research in the future. The visualization depends on a gene construct with a reporter gene, which can be used to transfer and cultivate the microorganisms, and a few endophytes that are not able to grow outside of the plant [181].

**Table 2:** Properties of the various microscopic techniques for the detection of microbial interaction (endophytic and pathogenic fungal interaction).

Detection System	Properties	Reference
Visualization	Interactions between plant and microorganism on the cellular level provides a more detailed understanding of the basic principles of how endophytes function.	[182]
Microscopy (contrast microscopy and interference microscopy (DIC))	Microscopy is a valuable technique for visualizing cells without staining, for example, endophytic colonization and microorganism interactions. Contrast microscopy and differential interference microscopy can be used to visualize the effects of the endophytes on the growth and hyphal morphology of the plant pathogen.	[183]
Enzymatic reporters genes	The reporter genes with enzymatic functions are used to visualize microorganism cells and gene expression. The use of reporters such as *lacZ* or *gfp* requires genetic transformation and an effective fast approach for studying plant–fungus interactions.	[184].
Scanning electron microscopy	SEM has a high resolution power, which can visualize the single cells of fungus, microcolonies, and the total microflora present as well as differentiate the morphological traits within endogenous communities of microorganisms.	[178]
Transmission electron microscopy (TEM).	A transmission electron microscope (TEM) is a microscope that uses a beam of particle electrons to look at a sample and create a larger image. A TEM can magnify objects up to 2 million times. To obtain a better idea of how small it is, consider how small the cell is. TEM has become very valuable in agriculture fields and studying the pattern of endophyte colonization within the intercellular and intracellular spaces of plant tissues. This is fitted with a Gatan Orius 1000 camera.	[185]
Epifluorescence microscopy	This is based on the presence of fluorescent compounds (i.e., proteins), which, after excitation with light of a certain wavelength, emits light of a longer wavelength due to energy loss during the process of absorption and excitation.	[186]
Confocal laser scanning microscopy (CLSM)	CLSM is used to study plant–pathogen interactions, which have revolutionized research into the role of selected molecules and cell components in pathogen infection strategies and plant defense responses. In combination with computational image analysis, it provides a powerful tool by which molecules, molecular interactions, and cell components can be localized and studied.	[187]
Auto-fluorescent	The use of different auto fluorescent proteins is an excellent tool to distinguish microorganisms from each other and to visualize their interactions between phytopathogenic fungi. Biocontrol agents will help to understand these interactions and facilitate the development of efficient biocontrol applications.	[188]

## 7. Role of Fungal Endophytes in Sustainable Agriculture

**Figure 3:**
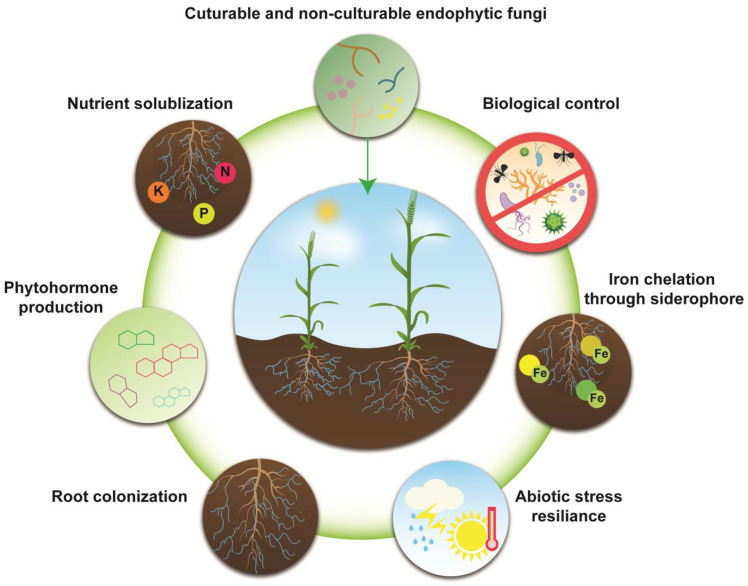
Potential roles of endophytes in agriculture.

The injudicious use of chemical pesticides has raised serious concerns of environmental pollution, toxic residual effect, resistance development in plant pathogens, and the non-target effect on beneficial microbes, which has shifted the interest of researchers in adopting eco-friendly alternatives to meet the growing demand of agricultural production [72,189,190,191,192]. Sustainable agriculture requires different strategies to increase the production of food while minimizing hazardous health effects (Figure 3) [193,194]. Endophytes play a vital role in sustainable agriculture by improving plant health and defending them from pathogen attacks [195,196]. Endophytes have attracted attention as biological fertilizers or phyto-remediation applications in agriculture [197]. Endophytic fungi exist in various plants, namely trees, herbs, grasses, and algae, and have established a symbiotic relationship with host plants [198,199,200]. This symbiotic relationship between plants and fungal endophytes produces biologically active substances (antifungal agents, antibacterial agents, insecticides, plant growth regulators, etc.), thereby improving the host plant’s tolerance to various biotic and abiotic stresses in nature [19,201,202]. 

Fungal endophytes act as biological control agents, with unique properties that enhance production and stress resistance in the host plant [203,204]. Several studies have shown that treating host plants with the selected endophytes can sensitize plants against disease causing pathogens [205,206]. Endophytes induce resistance in the host plants due to the production of defensive compounds, namely, alkaloids, flavonoids, terpenoids, quinones, chlorinated metabolites, iscoumarin derivatives, and phenolic compounds [153,207,208]. In addition, endophytes also induce systemic resistance by producing salicylic acid, HCN, siderophores, cell wall degrading enzymes and antifungal compounds, and direct antagonism to different plant pathogens [209,210]. The endophytes can produce plant growth promoting (PGP) active hormones in different crops such as gibberellin (GA), indole acetic acid (IAA), phosphate solubilization, and cytokinin [211,212]. 

Both climatic and environmental factors determine the nature of plant endophytes and their role in agriculture [213]. In order to maximize the benefits of fungal endophytes in crops, it is compulsory to understand the nature of microbial symbionts commonly present in a specific crop, the environmental growth conditions for their successful establishment in the host plant physiological system, their role in disease control, plant growth, and their ability to produce biologically active metabolites [214,215]. The main goal of agricultural researchers is to identify potential endophytes [216]. These potential endophytes are not only symbionts in a specific crop, but also perform well under various climatic conditions in which the crop is grown [217,218]. Crops with a proper endophyte proportion have stronger resistance and faster growth compared to crops lacking the potential endophytes [219]. The fungal endophytes also provide the host plant with enhanced resistance to abiotic and biotic stresses (i.e., drought, poor soil, and herbivory) [220,221]. The ability of endophytes to improve plant nutrition or secondary metabolites may lead to increased growth and stress resistance [222,223]. Many endophytes can protect plants from herbivorous hazards by insects and animals through production of secondary metabolites that are inedible or toxic to herbivores [224,225]. Much attention is being paid to endophytes to protect valuable crops against pathogen and insect attack [19,226]. The use of endophytes might potentially increase the crop yields [227]. 

There are numerous obstacles to the successful implementation of the use of endophytes in agriculture, although endophytes can confer many known benefits to host plants, conventional agriculture practices are still a priority. Modern agriculture mostly depends on chemical fertilizers and fungicides. The use of fungicides has an adverse effect on fungal endophytes, and chemical fertilizers reduce host plant dependence on their endophytic symbionts [228]. The interest and use of endophytes is increasing day by day to enhance agricultural production and they are considered as an important factor in sustainable agriculture. As humans have become more conscious about the negative effects of synthetic chemicals on the environment and beneficial microbes. Thus, fungal endophytes as biological control agents may become more important to the agricultural industry.

## 8. Conclusions and Future Perspectives

Endophytic fungus plays an indispensable role in plant physiology and functions of agricultural ecosystems. The fungal endophytes as MBCA provide an attractive, effective, and environmentally friendly method for controlling plant diseases. The widespread allocation of endophytes is well-known, with long-term results. Most fungal endophytes are closely related to their host plants and have obvious advantages (i.e., by producing many secondary metabolites/substances that can be used to control pathogens and enhanced plant growth). They can compete with plant pathogens to improve the immune status of plants, increase stress tolerance, and provide protection against pathogens and diseases. However, due to unfavorable environmental conditions, the stable growth and development of biological fungal agents under field conditions is still a challenge. Many fungal strains have been developed to effectively control plant diseases. To successfully use these biological agents, more in depth study will be needed. Understanding the appearance, identification, and function of important biological microorganisms in a specific environment is the first step to understanding their nature and mode of action in each environment. Due to the complexity of environmental factors and their effects on fungal endophytes, extensive research is needed to fully understand and characterize the interaction of MCBA with host plant pathogens. In the past few years, the development of different molecular detection technologies has revealed new and diverse information. The study of endophytic fungi provides important prospects for understanding the complex interactions within microbial communities and with the plant hosts. High-precision microscopy tools and the optimization of automated fluorescent proteins have made important contributions to understanding how these microorganisms interact with plants. These reasonable microscopy tools will promote this research in the future.

## Data Availability

Not applicable.
